# Surface plasmon resonance properties of silver nanoparticle 2D sheets on metal gratings

**DOI:** 10.1186/2193-1801-3-284

**Published:** 2014-06-05

**Authors:** Akira Baba, Keisuke Imazu, Akihito Yoshida, Daisuke Tanaka, Kaoru Tamada

**Affiliations:** Center for Transdisciplinary Research, Niigata University, 8050 Ikarashi 2-nocho, Nishi-ku, Niigata, 950-2181 Japan; Institute for Materials Chemistry and Engineering, Kyushu University, 6-10-1 Hakozaki, Higashi-ku, Fukuoka, 812-8581 Japan

**Keywords:** Propagating surface plasmon, Localized surface plasmon, Silver nanoparticles, Nanosheet

## Abstract

Grating-coupled propagating surface plasmons associated with silver-nanoparticle 2D crystalline sheets exhibit sensitive plasmonic resonance tuning. Multilayered silver-nanoparticle 2D crystalline sheets are fabricated on gold or silver grating surfaces by the Langmuir– Blodgett method. We show that the deposition of Ag crystalline nanosheets on Au or Ag grating surfaces causes a drastic change in propagating surface plasmon resonance (SPR) both in angle measurements at fixed wavelengths and in fixed incident-angle mode by irradiation of white light. The dielectric constant of the multilayered silver nanosheet is estimated by a rigorous coupled-wave analysis. We find that the dielectric constant drastically increases as the number of silver-nanosheet layers increases. The experimentally obtained SP dispersions of Ag crystalline nanosheets on Au and Ag gratings are compared with the calculated SP dispersion curves. The drastic change in the surface plasmon resonance caused by the deposition of Ag-nanoparticle 2D crystalline sheets on metal grating surfaces suggests the potential for applications in highly sensitive sensors or for plasmonic devices requiring greatly enhanced electric fields.

**Electronic supplementary material:**

The online version of this article (doi:10.1186/2193-1801-3-284) contains supplementary material, which is available to authorized users.

## Introduction

In recent years, surface plasmon resonance (SPR) phenomena have attracted considerable attention because of the extremely strong enhancement and confinement of electric fields near metal surfaces (Knoll 1998). Two kinds of optical excitations can occur at metal/dielectric interfaces, propagating surface plasmons and localized ones, but the geometries of the excitations differ. Propagating surface plasmons are excited at flat metal/dielectric surfaces under total internal reflection of irradiated light or at grating metal/dielectric interfaces (Raether 1988), while localized surface plasmons are excited at metal-nanoparticle/dielectric interfaces (Willets and Van Duyne 2007). Both excitations are sensitive to material adsorption events, which change the dielectric constant on the metal surface.

Recently, tuning of propagating SPR properties has attracted considerable interest from those seeking to develop applications for plasmonic devices (Obando and Booksh 1999; Baba et al. 2003; Baba et al. 2012;). In the attenuated total internal reflection format with the Kretschmann configuration, highly sensitive tuning of propagating surface plasmons has been obtained by changing the density of gold nanoparticles deposited on flat gold surfaces. This high sensitivity occurs because the dielectric constant of gold nanoparticles is much larger than that of organic materials (Li et al. 2009). The change in the dielectric constant on thin gold films was detected by the change in the SPR dip caused by the adsorption of gold nanoparticles modified with biomolecules on metal surfaces. This behavior leads to highly sensitive biosensor applications (He et al. 2000; Ito et al. 2007; Brolo 2012).

Tuning of the localized SPR spectrum associated with metal nanoparticles is also a key issue in applications of plasmonic devices (Jensen et al. 2000; Okamoto et al. 2013; Hsiao et al. 2008; Evans et al. 2007; Leroux et al. 2005; Leroux et al. 2008; Leroux et al 2009; Dintinger et al. 2006; Stockhausen et al. 2010; Yoshida et al. 2012). The localized SPR of metal nanoparticles is extremely sensitive to the particle size, composition, and dielectric constant of surrounding materials. Recently, assemblies of metal nanoparticles exhibited drastic changes in the plasmonic absorption wavelength because electromagnetic coupling between metal nanoparticles leads to an enhancement of the intense electric field, which depends on the distance between nanoparticles (Tao et al. 2007; Tao et al. 2008; Chen et al. 2008; Liz-Marzan 2006). This coupling causes two-dimensional nanoparticle nanostructured arrays to exhibit controllable plasmonic tuning (Courty 2010). Furthermore, using a layer-by-layer assembly technique, three-dimensional metal-nanoparticle supercrystals were created; these supercrystals show strong interlayer and intralayer near-field coupling (Lin et al. 2010).

**Figure 1:**
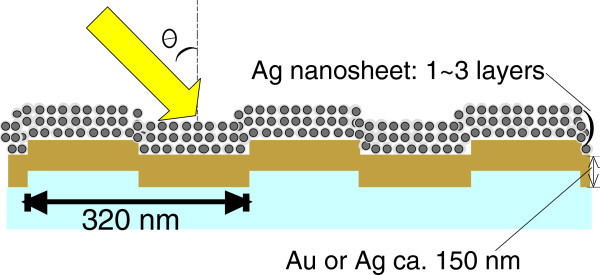
**Schematic of the nanosheet silver nanoparticles on a metal grating surface.**

Metal nanoparticles deposited on metal films exhibit distinct features on the basis of a dipole–dipole interaction model (Abe and Kajikawa 2006; Uchino and Kajikawa 2009; Uchimo et al. 2010; Hu et al. 2010). This leads to a large red shift in the plasmonic absorption spectrum. In particular, multilayered two-dimensional metal nanoparticles deposited on a metal surface showed extraordinary resonance changes. Furthermore, simultaneous propagating and localized SPR excitations have recently been reported (Yu et al. 2006; Live et al. 2009; Ding et al. 2011). Smith et al. showed that localized plasmon resonance could be observed when the density of gold nanoparticles increased on a flat gold surface, while propagating surface plasmons could also be observed in different visible wavelength regions by irradiating white light through a prism (Mock et al. 2012). However, to our knowledge, there is no report on the study of grating-coupled SPR properties with a multilayered two-dimensional metal-nanoparticle sheet.In this study, we report propagating SPR properties occurring by the deposition of 2D nanosheet silver-nanoparticle multilayers on a gold grating or on a silver grating surface. Figure 1 shows a schematic of the nanosheet silver nanoparticles on a metal grating surface. We found that the dielectric constant drastically increased as the number of nanosheet silver-nanoparticle layers increased. The experimentally obtained surface plasmon dispersion curves of Ag crystalline nanosheets on Au and Ag gratings were compared with calculated SP dispersion curves to study the increase in the dielectric constant of the multilayered Ag nanosheets.

## Experimental section

Silver nanoparticles were synthesized by thermal reduction of a silver acetate precursor in a melt of myristic acid, as described earlier (Keum et al. 2008). The resulting Ag nanoparticles (Ag core size, 4.8 nm ± 0.1 nm) capped by myristates (AgMy) were well-monodispersed. A stable 2D crystalline sheet (monolayer) spread at an air–water interface was transferred onto gold or silver grating substrates by the Langmuir–Blodgett (LB) technique. The interparticle distance of the 2D Ag nanosheet was estimated to be 1.9 nm by scanning electron microscope (SEM) imaging. Details of the fabrication of 2D Ag nanosheets can be found in previous papers (Toma et al. 2011). A polycarbonate blu-ray recordable disc (BD-R, Taiyo Yuden Co., Ltd.) was used as the grating substrate (Λ = 320 nm) because of the low-cost and simple technique (Baba et al. 2011; Kaplan et al. 2009; Singh and Hillier 2006). The BD-R was cut into pieces, which were then immersed in nitric acid to remove the dye layer from the grating side. The cleaned pieces were coated with a layer of gold or silver (thickness ~150 nm) by vacuum evaporation at a deposition rate of 1.0/sec at 6.7 × 10^-4^ Pa. (ULVAC, VPC-400). The grating samples were mounted on a *θ*-2*θ* goniometer. A halogen lamp as the white light source or a HeNe laser was used for the excitation of SPs. P-polarized light was collimated by objective lens and irradiated on the grating samples. The reflected zero-order light was detected by a spectrometer. SPR excitation experiments were carried out at a fixed incident angle as a function of wavelength or at a fixed wavelength as a function of incident angle. Grating-coupled SPR modeling was performed on G-Solver (Grating Solver Development, Co.) using rigorous coupled-wave analysis.

## Results and discussion

**Figure 2:**
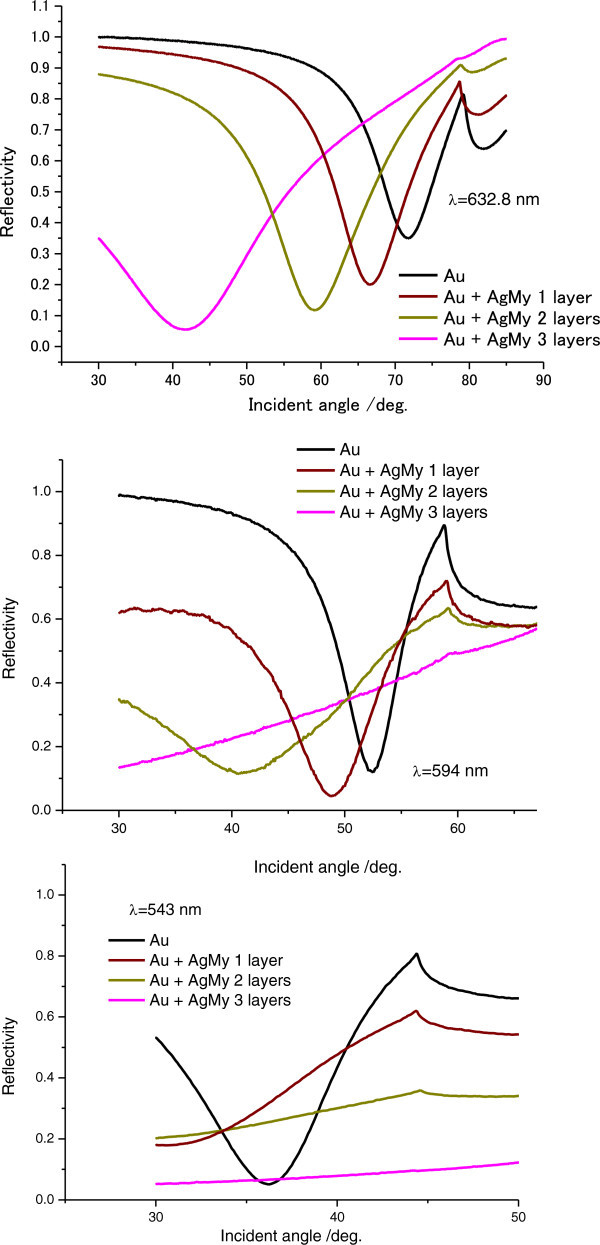
**Grating-coupled angular SPR reflectivity curves for multilayered AgMy nanosheets on a gold grating film measured at 632.8 nm, 594 nm, and 543 nm.**

**Figure 3:**
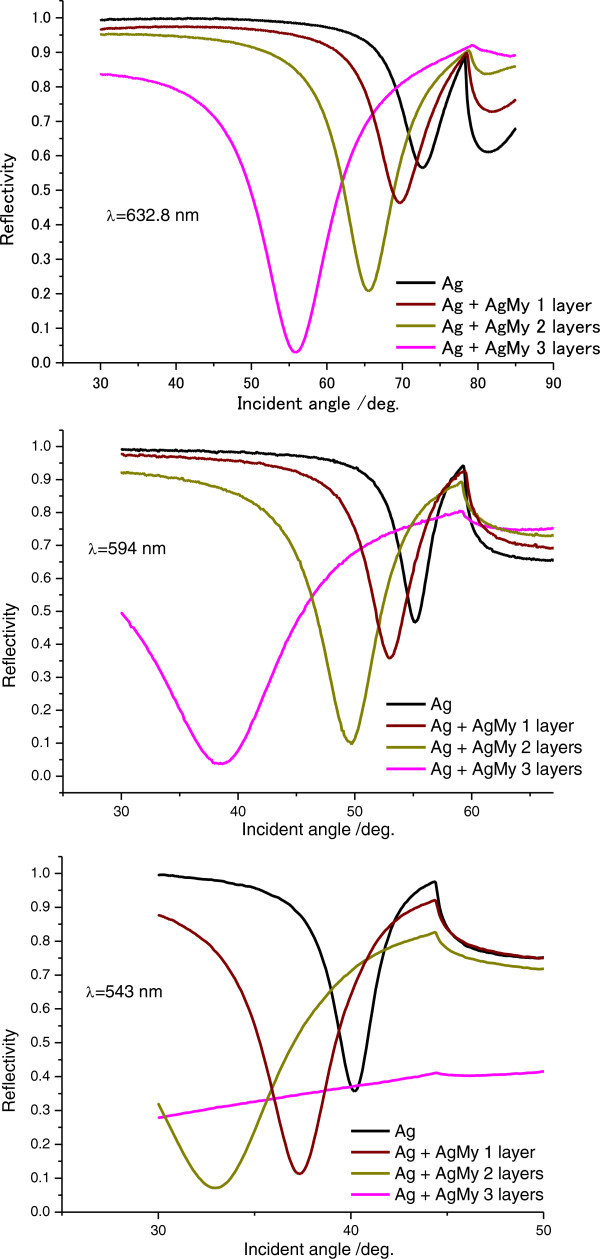
**Grating-coupled angular SPR reflectivity curves for multilayered AgMy nanosheets on a silver grating film measured at 632.8 nm, 594 nm, and 543 nm.**

**Figure 4:**
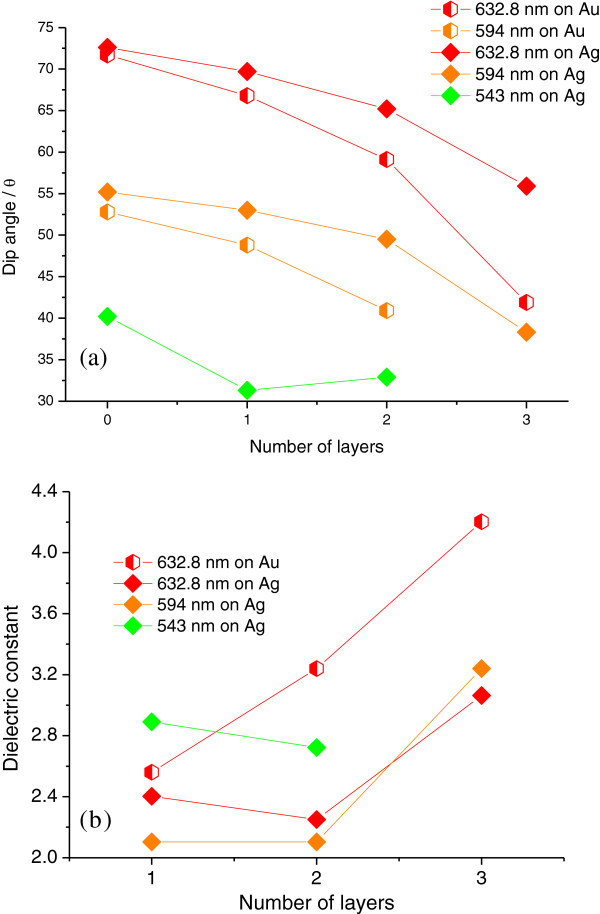
**Plots obtained from SPR reflectivity curves on gold and silver grating films.** **(a)** Shifts in SPR dip angle. **(b)** Dielectric constant of AgMy nanosheet as a function of the number of layers.

Figure 2 shows grating-coupled angular SPR reflectivity curves from multilayered Ag nanoparticles (Ag core size, 4.8 nm ± 0.1 nm) capped by 2 nm-thick myristates (AgMy) nanosheets on gold grating films measured at 632.8 nm, 594 nm, and 543 nm. The figure shows that, at each wavelength, the dip angles of the reflectivity curves shift toward lower angles as the number of AgMy nanosheet layers increases. For angular SPR properties on silver grating films, Figure 3 also shows that the dip angle nonlinearly shifts to lower angles, and for three layers, the shift in the dip angle is large. The plots of the angles on both gold and silver gratings in Figure 4(a) clearly show the large shift for three layers. In theoretical simulations, if the dielectric constant is constant, the dip angle would approximately monotonically decrease as the thickness of deposited materials increases on metal gratings (See Additional file 1: Figure S1). However, Figure 4(a) shows that the dip angles decreases nonlinearly with the number of AgMy nanosheet layers. This indicates that the dielectric constant of the AgMy nanosheet changes as the number of layers change.

Modeling the SPR dip produced by the nanosheets confirmed an increase in the dielectric constant with the number of layers. In calculation, the dielectric constant of each layer could be adjusted to match the shift because the thickness of AgMy nanosheets was obtained in previous work and was assumed to be constant. Figure 4(b) contains plots obtained by rigorous coupled-wave analysis using G-Solver. The calculations were carried out using an assumed thickness (9 nm) for each nanosheet layer, because our previous study showed AgMy nanosheet was well formed to be a monolayer (Okamoto et al. 2013). As Figure 4(b) shows, the dielectric constant at 632.8 nm did indeed increase from ϵ = 2.56 for one nanosheet layer to ϵ = 4.2 for three nanosheet layers on the gold grating surface. For the silver grating, the dielectric constant suddenly increased at three AgMy nanosheet layers. Since the deposited AgMy nanosheets were exactly the same for each layer, this change in the dielectric constant is surprisingly large. To our knowledge, this is the first report that provides an estimate of the dielectric constant of a metal-nanoparticle nanosheet multilayer. In our previous study, the large increase in the dielectric constant of gold nanoparticles was attributed to plasmonic interactions between adjacent nanoparticles when the nanoparticles were closely packed (Li et al. 2006). However, because the AgMy nanosheet is already closely packed on the Ag nanoparticle 2D crystalline sheet (Okamoto et al. 2013; Yoshida et al. 2012; Toma et al. 2011), the increase in the dielectric constant found in this study mostly due to interactions either between intralayer nanoparticle nanosheets, or between nanosheets and the metal grating films. Moreover, the strong interference effect due to the nanometer optical coatings of the strongly absorbing material on metalallic materials, might effect the large shift (Kats et al. 2013).

In our previous report, a red shift in the LSPR absorption peak was observed when the AgMy nanosheet was deposited on flat metal surfaces, while there was no change in the absorption peak on a glass substrate (Toma et al. 2011). This behavior might affect the increase in dielectric constant at each wavelength. Another possibility is interactions between localized surface plasmons and propagating surface plasmons. Propagating surface plasmons on both Au and Ag gratings are clearly observed for up to three nanosheet layers at 632.8 nm. However, the dip due to propagating surface plasmons becomes broader and the reflectivity is very low not only at around the SPR dip angle but also in the angle region higher than the critical angle, especially at 543 nm. This is especially obvious for three layers on both the Au and Ag gratings. As the number of layers increases, the localized plasmon peak shifts to longer wavelength (Okamoto et al. 2013) and becomes closer to the observed wavelength of propagating SPR, resulting the decrease of the reflectance at around the wavelength, hence the broadened and lowered reflectance SPR curves might be due to coexcitation of LSPR and propagating SPR, resulting in confined energy near the surface in the broad-angle region.

**Figure 5:**
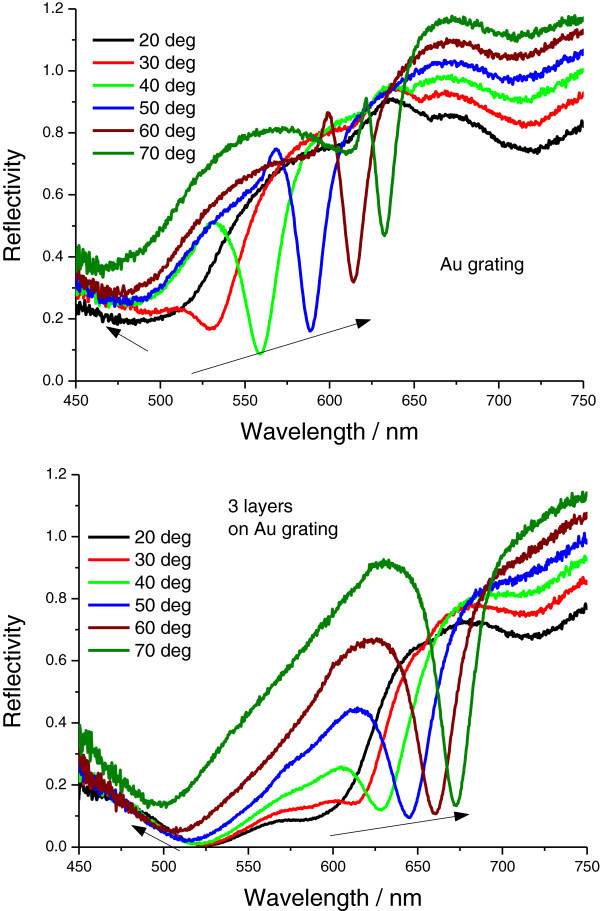
**SPR reflectivity curves from a bare Au grating (top) and from three AgMy nanosheet layers on the Au grating (bottom) at fixed angles from 20° to 70° as a function of wavelength.**

**Figure 6:**
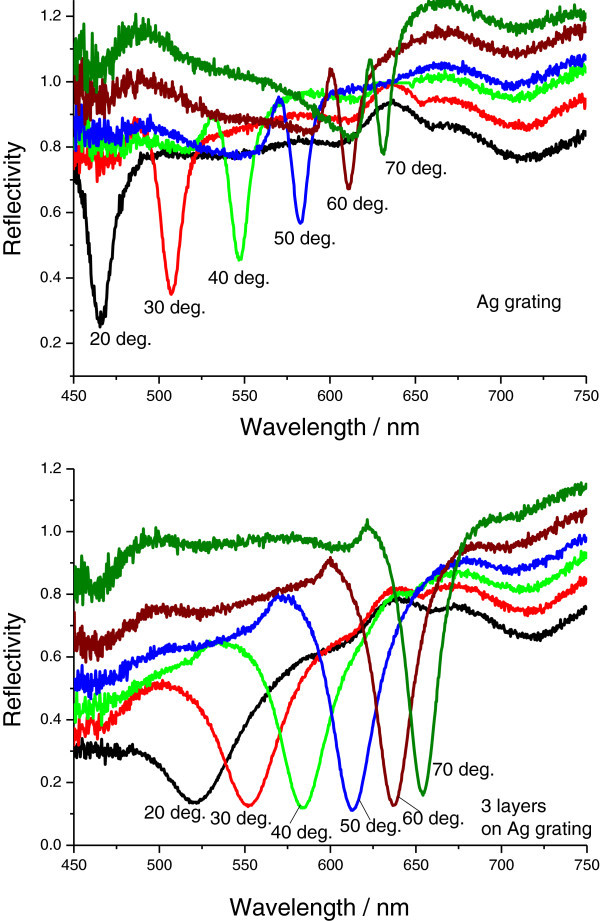
**SPR reflectivity curves from a bare silver grating (top) and from three AgMy nanosheet layers on the Ag grating (bottom) at fixed angles from 20° to 70° as a function of wavelength.**

**Figure 7:**
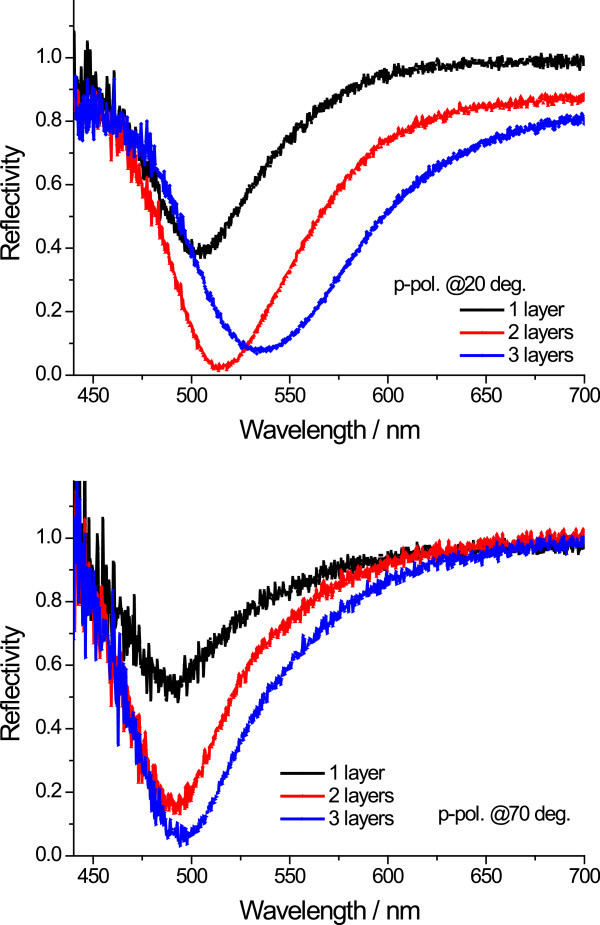
**Reflectance curves from one to three AgMy nanosheet layers on the flat Au at fixed angles of 20° and 70° as a function of wavelength at p-polarization.**

To further study the unusual changes in dielectric constants, we measured SPR excitations at fixed angles from 20° to 70° as a function of wavelength for bare Au grating and for three AgMy nanosheet layers on the Au grating, as shown in Figure 5. In this figure, the sharp dip moves to longer wavelengths as the incident angle increases, while the shallow dip shifts to shorter wavelengths, especially for three layers of AgMy nanosheets. Both dips were shifted to longer wavelengths as the AgMy nanosheets were deposited on the Au grating surface. In the case of Ag grating (Figure 6), only one sharp dip for each incident angle was observed; this dip also shifted to higher wavelengths as the dip angle increased and as AgMy nanosheets were deposited on the Ag surface. To study the effect of LSPR on the metal films, we measured reflectance curves from one to three AgMy nanosheet layers on the flat Au at fixed angles of 20° and 70° as a function of wavelength at p-polarization. As shown in Figure 7, the reflectance dip at 20° due to the LSPR absorption shifts to higher wavelength as the number of AgMy nanosheet increases, while the dip wavelength at 70° is almost constant. The trend of the wavelength change on flat Au corresponds well with the shallow dips in Figure 5, confirming that the origin of the shallow dips is due to the LSPR absorption. This indicates that the low reflectivity in the range from 450 to 650 nm (in Figure 5) should be due to the LSPR and propagating surface plasmon co-excitations. The broadened reflectivity curves of 3 AgMy nanosheets on Ag at 20° in Figure 6 should also be due to the effect of the LSPR besides the propagating SPR excitation. As seen in supporting informations SI2-SI5, the wavelength shift was observed only on the metal film, and the angle dependence of the dip wavelength was observed only in the case of the irradiation of p-pol. light on the metal film. There is a possibility that the shift of LSPR absorption affects the dip wavelength in the reflection mode.^12^ It is interesting to note that the dip clearly shows angle dependence for three AgMy layers, indicating some interaction between the AgMy nanosheet and the Au metal surface. Recently, Kats et al reported the wavelength shift and angle dependence by the deposition of highly absorbing nanomaterials on Au surface at p-pol. light irradiation, which was originated from Fabry-Perot-type interference (Kats et al. 2013). In our case, because the metal nanoparticles has high dielectric constants with strong absorption due to LSPR, the similar wavelength shift and angle dependence might be generated by the deposition of multilayered AgMy nanosheet.

**Figure 8:**
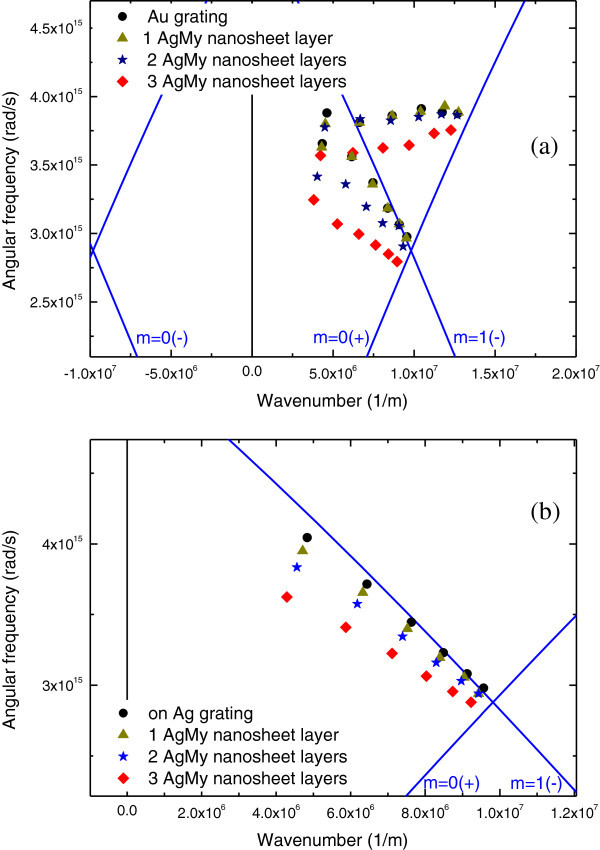
**Experimental dip angles (symbols) and calculated SP dispersion branches (solid lines).** **(a)** on the Au grating. **(b)** on the Ag grating.

For each dip angle and wavelength in Figures 5 and 6, we plotted the corresponding SP dispersion (symbols) as shown in Figure 8. Calculated SP dispersion relations on the silver and gold gratings for m = 0(+) and m = +1(−) modes are also shown (solid curves). The SP dispersion can be obtained from the SP excitation condition defined as1

Here, *Λ* is the diffraction grating pitch, *λ* is the wavelength, *m* is the diffraction order, and ϵ_m_(ω) is the wavelength-dependent dielectric constant of silver given by the classical Drude’s free-electron model. As shown in Figure 8, the wavelength, which corresponds directly to angular frequency, becomes shorter as the wavenumber of the SP dispersion branch for the m = 1(−) mode decreases. Here, the wavenumber of the SP dispersion branch corresponds directly to the incident angle. The SP dispersion of the Au grating almost corresponds to the m = 1(−) mode, although some discontinuities and small errors are observed from the calculated curves. The plots clearly indicate that the SP dispersion for two and three AgMy layers show a large shift from the bare Au grating SP dispersion. Conversely, the plot for the shallow dips did not correspond to any theoretical SP dispersion curve. This indicates that the origin of the shallow dip in Figure 4 is not responsible for the propagating SP excitation on the Au grating surface. This is reasonable that the dips originated from LSPR absorption were observed on flat Au as discussed in Figure 7. For the Ag grating surface, the experimental SP dispersion data correspond to the theoretical SP dispersion curve for the m = 1(−) mode. Similarly, a large shift in the SP dispersion plot was observed for three AgMy nanosheet layers, indicating plasmonic interactions.

## Conclusions

We studied the SP excitation properties of Ag crystalline nanosheets on Au and Ag grating surfaces, and found a drastic change in SP excitation from angular measurements at fixed wavelength and from measurements at fixed incident angle under irradiation with white light. The SPR dips were drastically shifted when Ag crystalline nanosheets were deposited on the grating surfaces. The experimentally obtained SP dispersion data of the Ag crystalline nanosheets on Au and Ag gratings were compared to calculated SP dispersion curves. A large shift in the wavelength or dip angle by the deposition of Ag nanoparticle 2D crystalline sheets on a metal grating surface based on the drastic change in the surface plasmon resonance suggests the potential for applications in highly sensitive sensors or for plasmonic devices requiring greatly enhanced electric fields.

## Electronic supplementary material

Additional file 1: **Supporting Information.** (PDF 384 KB)
