# Supplementary material for: Surface plasmon resonance properties of silver nanoparticle 2D sheets on metal gratings
Source: Springerplus. 2014 Jun 5;3:284. doi: 10.1186/2193-1801-3-284 (PMC4059854; doi:10.1186/2193-1801-3-284)
Supplement: Supplementary file 1 — Additional file 1: Supporting Information. (PDF 384 KB) [file 40064_2014_999_MOESM1_ESM.pdf]

Supporting Information

Surface Plasmon Resonance Properties of Silver Nanoparticle 2D Sheets on Metal Gratings

Akira Baba[†,]*, *Keisuke Imazu*[‡], *Akihito Yoshida*[‡], *Daisuke Tanaka*[‡], *Kaoru Tamada*[‡,]*

[†]Center for Transdisciplinary Research, Niigata University, 8050 Ikarashi 2-nocho, Nishi-ku, Niigata 950-2181, Japan

[‡]Institute for Materials Chemistry and Engineering, Kyushu University, 6-10-1 Hakozaki, Higashi-ku, Fukuoka 812-8581, Japan

E-mail: ababa@eng.niigata-u.ac.jp, tamada@ms.ifoc.kyushu-u.ac.jp

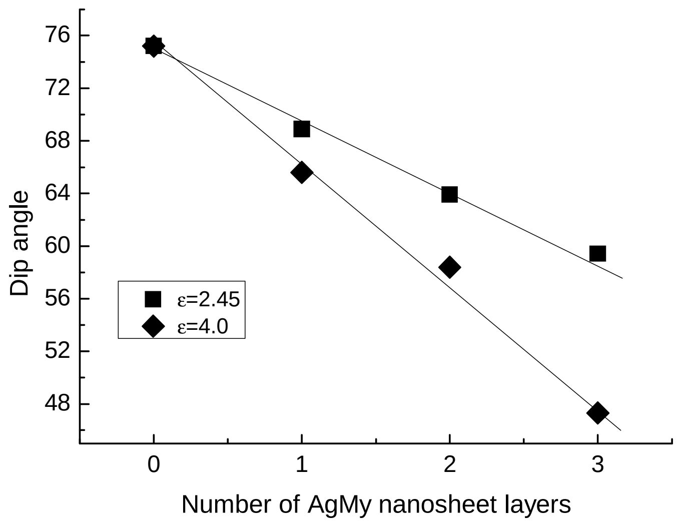

Figure S1. Shifts in dip angle on a silver grating film as a function of the number of AgMy nanosheet layers. Dielectric constant of AgMy nanosheet layers was assumed to be constant (ε=2.45 or 4.0)

In theoretical simulations, if the dielectric constant is constant, the dip angle approximately monotonically decrease as the thickness of deposited materials increases on metal gratings.

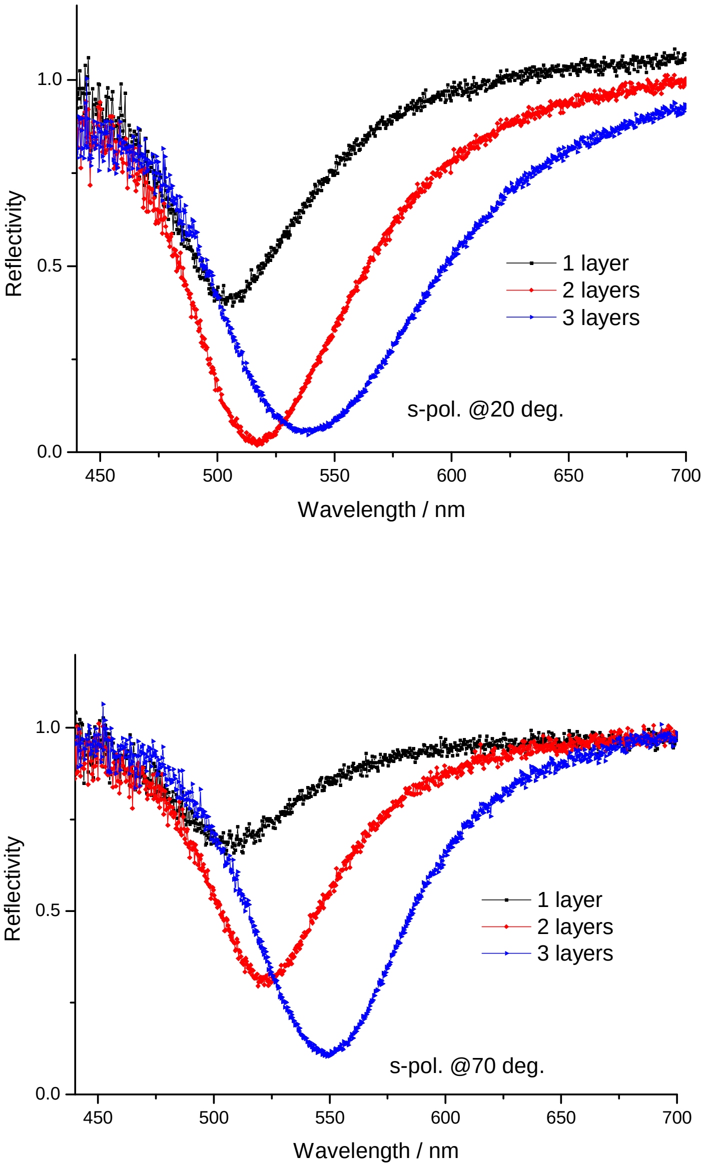

s-pol. on Au

Figure S2. Reflectance curves from one to three AgMy nanosheet layers on the flat Au at fixed angles of 20° and 70° as a function of wavelength at s-polarization.

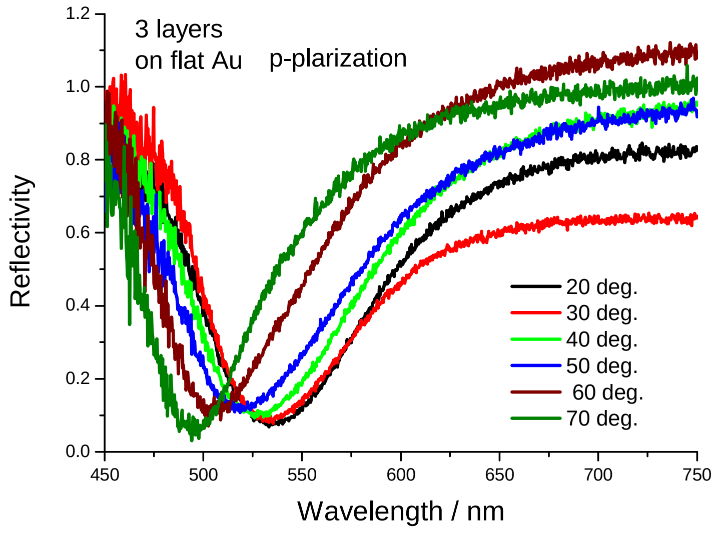

p-pol.

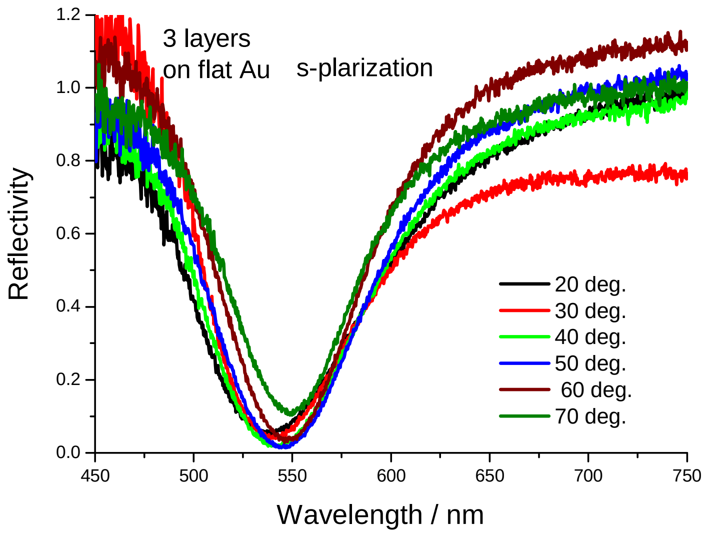

s-pol.

3 layers on Au

Figure S3. Reflectance curves from three AgMy nanosheet layers on the flat Au at fixed angles from 20° to 70° as a function of wavelength.

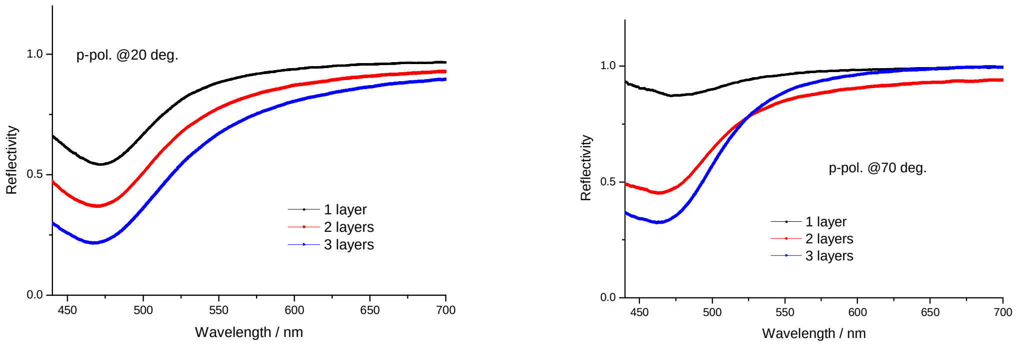

p-pol. on glass

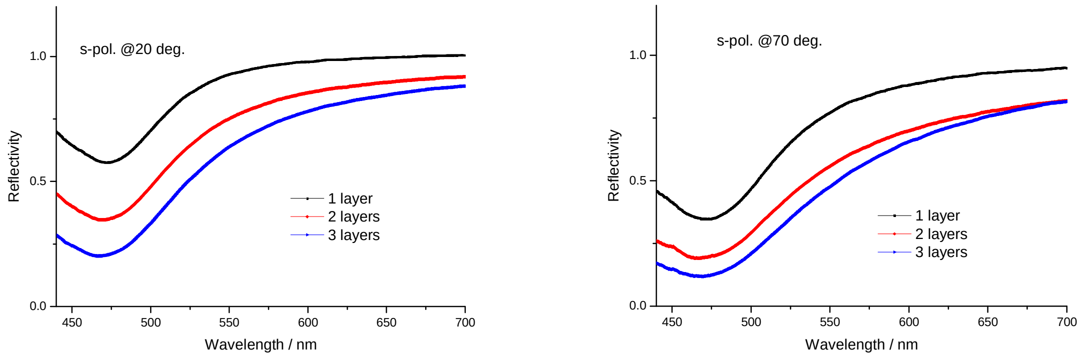

s-pol. on glass

Figure S4. Reflectance curves from one to three AgMy nanosheet layers on the flat glass substrates at fixed angles of 20° and 70° as a function of wavelength at p and s-polarization.

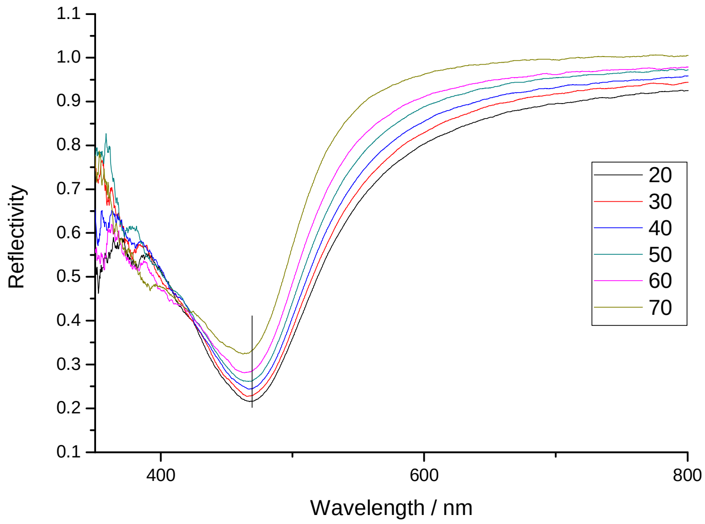

p-pol.

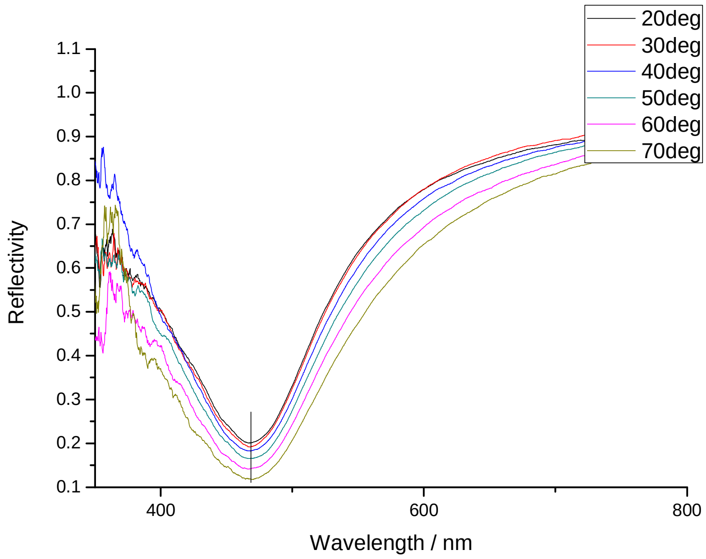

s-pol.

3 layers on glass

Figure S5. Reflectance curves from three AgMy nanosheet layers on the flat glass substrates at fixed angles from 20° to 70° as a function of wavelength.
